# P-loop mutations and novel therapeutic approaches for imatinib failures in chronic myeloid leukemia

**DOI:** 10.1186/1756-8722-1-15

**Published:** 2008-10-01

**Authors:** Shundong Cang, Delong Liu

**Affiliations:** 1 Division of Hematology/Oncology, New York Medical College, Valhalla, NY 10595, USA

## Abstract

Imatinib was the first BCR-ABL-targeted agent approved for the treatment of patients with chronic myeloid leukemia (CML) and confers significant benefit for most patients; however, a substantial number of patients are either initially refractory or develop resistance. Point mutations within the ABL kinase domain of the BCR-ABL fusion protein are a major underlying cause of resistance. Of the known imatinib-resistant mutations, the most frequently occurring involve the ATP-binding loop (P-loop). *In vitro *evidence has suggested that these mutations are more oncogenic with respect to other mutations and wild type BCR-ABL. Dasatinib and nilotinib have been approved for second-line treatment of patients with CML who demonstrate resistance (or intolerance) to imatinib. Both agents have marked activity in patients resistant to imatinib; however, they have differential activity against certain mutations, including those of the P-loop. Data from clinical trials suggest that dasatinib may be more effective vs. nilotinib for treating patients harboring P-loop mutations. Other mutations that are differentially sensitive to the second-line tyrosine kinase inhibitors (TKIs) include F317L and F359I/V, which are more sensitive to nilotinib and dasatinib, respectively. P-loop status in patients with CML and the potency of TKIs against P-loop mutations are key determinants for prognosis and response to treatment. This communication reviews the clinical importance of P-loop mutations and the efficacy of the currently available TKIs against them.

## Background

Chronic myeloid leukemia (CML) accounts for approximately 20% of all adult leukemias in the United States [1]. Progression of CML is generally described as a three-phase process, beginning in a mostly asymptomatic chronic phase (CP), progressing to an intermediate accelerated phase (AP) and followed by a usually terminal blast phase (BP) [1]. Left untreated, CML usually progresses from CP to BP over a period of 3 to 5 years [1].

CML is characterized by the Philadelphia chromosome, which results from a genetic translocation between chromosomes 9 and 22 [2,3]. This translocation results in fusion of the BCR and ABL genes, which code for a constitutively active BCR-ABL tyrosine kinase [4,5]. The activity of this BCR-ABL tyrosine kinase, including its anti-apoptotic effects, underlies the pathophysiologic basis of CML [6-8].

Modern treatment of CML relies upon tyrosine kinase inhibitors (TKIs) directed against BCR-ABL. Imatinib (Gleevec^®^, Novartis Pharmaceuticals Corporation, East Hanover, NJ, USA) was the first TKI approved for the treatment of CML and is the current first-line treatment. Approval of this agent was based on data from the International Randomized Study of Interferon and STI571 (IRIS) [9]. While most patients benefit from imatinib treatment, a substantial number either are initially refractory (primary resistance) or develop resistance during the course of treatment (acquired resistance). As a result of primary resistance to imatinib, 24% of patients in IRIS failed to achieve a complete cytogenetic response (CCyR) after 18 months [9]. Additionally, secondary resistance manifested as progression to advanced phases in 7% of patients and as relapsed disease in approximately 17% of patients [10].

Several underlying mechanisms of imatinib resistance have been identified. One major cause is the presence of point mutations within the ABL kinase domain of BCR-ABL. Such mutations inhibit the ability of imatinib to bind to BCR-ABL by corrupting the binding sites or preventing the kinase domain from assuming the inactive conformation required for imatinib binding [11]. Point mutations develop in approximately 35% to 70% of patients displaying resistance to imatinib, either spontaneously or through the evolutionary pressure of imatinib [12,13].

More than 40 distinct resistance-conferring mutations have been detected; the majority fall within four regions of the kinase domain: the ATP-binding loop (P-loop) of the ABL kinase domain, the contact site, the SH2 binding site (activation loop), and the catalytic domain [14]. Approximately 85% of all imatinib-resistant mutations are associated with amino acid substitutions at just seven residues (P-loop: M244V, G250E, Y253F/H and E255K/V; contact site: T315I; and catalytic domain: M351T and F359V) [15]. The most frequently mutated region of BCR-ABL is the P-loop, accounting for 36% to 48% of all mutations [12,13].

The importance of P-loop mutations is further underlined by *in vitro *evidence suggesting that these mutations are more oncogenic with respect to unmutated BCR-ABL as well as other mutated variants [16]. In various biological assays, P-loop mutants Y253F and E255K exhibited an increased transformation potency relative to unmutated BCR-ABL. Overall, the relative transformation potencies of various mutations were found to be as follows: Y253F > E255K > native BCR-ABL ≥ T315I > H396P > M351T. Transformation potency also correlated with intrinsic BCR-ABL kinase activity in this study.

Two agents are currently approved for second-line treatment of patients with CML who demonstrate resistance (or intolerance) to imatinib: dasatinib and nilotinib [17,18]. While both agents have marked activity in patients resistant to imatinib, they are differentially efficacious against certain mutations, including those of the P-loop. Data from clinical trials suggest that dasatinib may be more effective than nilotinib in treating patients harboring P-loop mutations. This communication reviews the clinical importance of P-loop mutations and the efficacy of the currently available TKIs against them.

### P-loop mutations and the response to imatinib

The mutations conferring resistance to imatinib have been well characterized [11]. The mutation analysis have been done using denaturing high-performance liquid chromatography and direct sequencing [15]. In the GIMEMA study, mutations were found in 43% of evaluable patients (127 of 297 patients). Among them, mutations were found in 27% with chronic phase patients, 52% of AP patients, and 75% of myeloid BC, and 83% lymphoid BC/Ph+ ALL [15]. The frequency of p-loop mutations clearly increases in accelerated phase and blast crisis as well as with disease duration [11,15]. Therefore patients with CML in these phases tend to develop imatinib-resistant mutations. Selection of resistant clones during therapy and clonal cytogenetic evolution with longer duration may be responsible for the development and expansion of the resistant clones with the mutations. These mechanisms argue against high-sensitivity mutation screening of all CML patients before therapy. It is prudent to do mutation analysis for patients who failed imatinib or have advanced CML.

**Figure 1:**
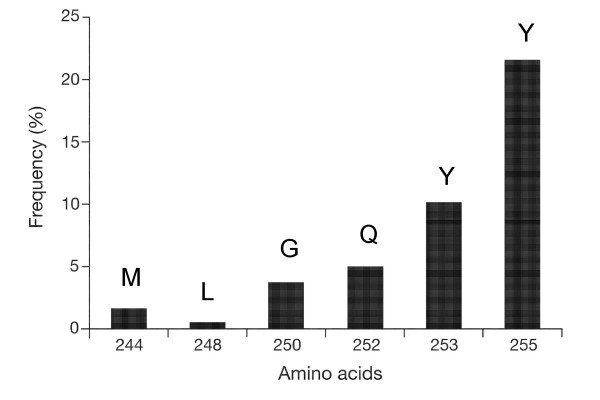
**Frequency of BCR-ABL P-loop mutations detected in 177 clinical specimens.** The positions of the P-loop amino acid residues were indicated. M-methionine; L-leucine; G-glycine; Q-glutamine; Y-tyrosine. (adapted from ref. [19]).

As mentioned previously, the most widely studied and clinically dominant mechanisms of imatinib resistance involve acquired point mutations within the kinase domain of BCR-ABL. BCR-ABL mutants can be grouped based on imatinib sensitivity: sensitive (IC_50 _≤ 1000 nM); intermediately sensitive (IC_50 _≤ 3000 nM; ie, M244V, G250E, Q252H, F317L and E355G); and insensitive (IC_50 _> 3000 nM; ie, Y253F/H, E255K/V and T315I). The various mutations occur at different frequencies and confer different levels of imatinib resistance (Fig. 1) [19].

The sensitivity of imatinib to many of these point mutations has been studied *in vitro *(Table 1). BCR-ABL mutated at the P-loop is 70-fold to 100-fold less sensitive to imatinib compared with native BCR-ABL. The presence of these mutations also has been associated with poor prognosis for patients receiving imatinib. Indeed, before the availability of second-line TKIs, patients with P-loop mutations treated with imatinib alone experienced reduced response and survival rates [12,13,20]. For example, Brandford *et al*. found that in patients with CP and AP CML, P-loop mutations were associated with death in 12 of 13 patients (92%; median survival of 4.5 months) vs. 3 of 14 patients with mutations outside of the P-loop (21%; median survival of 11 months) [12]. Similarly, Soverini *et al*. found that among CP patients with P-loop mutations, 8 of 9 patients experienced disease progression to AP or BC after a median of 9 months from mutation detection and 12 months from the onset of imatinib [20]. Only 3 of 9 patients with mutations outside of the P-loop experienced disease progression to AP or BC. Deaths also were reported more frequently with P-loop mutations (6 of 9 patients compared with 1 of 9 patients). Similarly, Nicolini *et al*. observed that among 89 patients with CML (64% CP) after a median follow-up of 39.2 months since imatinib initiation, overall survival was significantly worse for those with P-loop mutations (28.3 months) compared with other mutations (not reached) [21]. Furthermore, a recent study found that P-loop mutations were detectable 2.8 months before the development of resistance in patients taking imatinib compared with 6.3 months for T315I mutations, 10.8 months for M351T mutations, 2.9 months for A-loop mutations and 8.7 months for other mutations [22]. Additionally, of the 7 patients with mutations that were not detectable before relapse, 6 (86%) had P-loop mutations. Taken together, this information highlights the increased rate of progression associated with P-loop mutations. Because the appearance of such mutations seems to indicate impending relapse and resistance to imatinib, earlier detection may provide clinical benefit for patients by prompting earlier reconsideration of therapeutic interventions [22].

**Table 1:** Sensitivity of Bcr-Abl kinase domain P-loop mutants to imatinib, nilotinib and dasatinib

		**Ba/F3 cellular proliferation IC_50 _value**		
		
		**Imatinib (nM)**	**Nilotinib (nM)**	**Dasatinib (nM)**
**M244V**	**P-loop**	*2000*	38	1.3
**G250E**	**P-loop**	*1350*	48	1.8
**Q252H**	**P-loop**	*1325*	70	3.4
**Y253F**	**P-loop**	**3475**	125	1.4
**Y253H**	**P-loop**	**> 6400**	*450*	1.3
**E255K**	**P-loop**	**>5000**	*200*	5.6
**E255V**	**P-loop**	**> 6400**	*430*	11

Imatinib: sensitive (≤ 1000 nM), intermediate (≤ 3000 nM), insensitive (> 3000 nM). Nilotinib: sensitive (≤ 150 nM), intermediate (= 150 nM to 450 nM), insensitive (> 2000 nM). Dasatinib: sensitive (≤ 5 nM), intermediate (≤ 5 nM to 11 nM), insensitive (> 11 nM). (adapted from Ref. 11).

Sensitive

Intermediate sensitivity

Insensitive

In contrast, other studies in which patients were permitted to switch to second-line treatment showed no significant prognostic differences between patients carrying P-loop mutations vs. those with other mutations within BCR-ABL [13,23]. This result may be due to the availability of newer TKI therapies with greater activity against mutations of the P-loop for imatinib-resistant patients (Table 2). Alternatively, it is possible that the results of this study were influenced by differences in the specific P-loop mutations harbored by patients included in each study and/or differences in definition of the P-loop mutations may have contributed to different outcomes. With regard to the latter, Jabbour *et al*. defined P-loop mutations as those at residues 244 through 255 [13], while others included only mutations at residues 250 through 255 [12,20] or 248 through 255 [21].

**Table 2:** Efficacy of dasatinib and nilotinib in patients with CP CML harboring specific mutations

	CCyR rates, n/N (%)	
	
	Dasatinib	Nilotinib
Any mutation	158/369 (43)	18/77 (23)
P-loop mutations	61/141 (43)	NR
L248V	NR	0/2 (0)
G250E	19/51 (37)	1/4 (25)
Y253F/H	12/23 (52)	0/8 (0)
E255K/V	8/24 (33)	0/6 (0)
T315I	0/20 (0)	0/4 (0)
F317L	1/14 (7)	NR*^†^
F359C/V	14/27 (52)	0/10 (0)*

Dasatinib data are based on 1,093 patients with CP CML enrolled in clinical trials with dasatinib [36]. Nilotinib data are based on 280 patients with CP CML enrolled in a phase 2 clinical trial with nilotinib [42].

*(Adapted from Ref. [45]).

^†^Considered a nilotinib-sensitive mutation (18 of 45 patients harboring nilotinib-sensitive mutations achieved CCyRs) [45].

CP: chronic phase. CML: chronic myeloid leukemia. CCyR: complete cytogenetic response. NR: not reported.

As with all BCR-ABL mutants, P-loop mutations are detected more frequently in late-stage disease. Interestingly, advanced CML is an independent factor associated with their development [12,13,15]. When Soverini *et al*. examined the frequency and distribution of mutations according to disease phase at the time of diagnosis, they found that 52% of patients with AP CML and 75% of those with BP CML had mutations, compared with only 27% of patients in CP [15]. They also noticed a preferential association of P-loop and T315I mutations with advanced phase disease. This is not surprising, as supporting pre-clinical evidence has shown the increased oncogenic potential of P-loop mutations [16].

### Dasatinib

Dasatinib is a potent, orally active, dual BCR-ABL/Src-family kinase inhibitor [24]. Initial approval of dasatinib was based on data from the START (SRC/ABL Tyrosine kinase inhibition Activity: Research Trials of dasatinib) program, a series of multicenter, open-label phase 2 clinical trials in imatinib-resistant or -intolerant patients with CML or Philadelphia chromosome-positive acute lymphoblastic leukemia (Ph+ ALL). In the START-C trial, dasatinib was evaluated in patients with CP CML who were resistant or intolerant of imatinib [25]. A recent update to this trial showed that following 24 months of treatment, dasatinib 70 mg twice daily was associated with a high rate of durable cytogenetic responses in patients with CP CML who were resistant or intolerant to imatinib (Table 3) [26]. After 24 months of treatment, the major cytogenetic response (MCyR) rate was 62% and responses were durable with 88% of patients maintaining their response. The CCyR rate was 53% and the major molecular response was 47%. Additionally, at 24 months, progression-free survival was 80% (75% in imatinib-resistant and 94% in imatinib-intolerant patients) and overall survival was 94% (92% in imatinib-resistant and 100% in imatinib-intolerant patients) [26]. Marked activity also was noted in advanced disease [27,28].

**Table 3:** Clinical responses to dasatinib and nilotinib in CML treatment

Patients	McyR(%)		CCyR (%)		PFS (%)		OS (%)	
	DAS	NIL	DAS	NIL	DAS	NIL	DAS	NIL

Imatinib-resistant	55	48	NR	30	75	NR	92	NR
Imatinib-intolerant	78	47	78	35	94	NR	100	NR
All patients	62	48	53	31	80	NR	94	NR

MCyR: major cytogenetic remission; CCyR: complete cytogenetic remission; PFS: progression free survival; OS: overall survival; DAS: Dasatinib; NIL: nilotinib; NR: not reported [26,42].

Dasatinib was initially approved at a dosage of 70 mg twice daily (with or without food) for all stages of CML. The label has recently been updated such that 100 mg once daily is now the recommended regimen in CP CML [17]. This update was based on an open-label, dose-optimization study in which patients were randomized (1:1:1:1) to receive one of four dasatinib regimens: 100 mg once daily, 50 mg twice daily, 140 mg once daily or 70 mg twice daily [29]. The 100-mg, once-daily dosage demonstrated equivalent efficacy compared with the previously recommended 70-mg twice-daily dosage and was associated with fewer grade 3/4 adverse events (AEs; 30% vs. 48%, respectively) [29]. Most significantly, the 100-mg dose was associated with lower rates of pleural effusions (7% vs. 16%) and grade 3/4 thrombocytopenia (22% vs. 37%). Most other AEs were mild to moderate (grades 1/2) in severity and tended to resolve either spontaneously or with supportive care. Additionally, fewer discontinuations and dose modifications occurred in the 100-mg once-daily arm compared with the 70-mg twice-daily arm. Following results of this trial, the recommended starting dose of dasatinib for imatinib-resistant or -intolerant patients with CP CML was changed to 100 mg once daily [17]. The 70-mg twice-daily dosage remains the recommended starting dosage for patients with advanced phase disease (AP/BP CML or Ph+ ALL).

The marked activity of dasatinib in patients resistant to imatinib can be understood by noting its mechanism of action. Due to structural differences from imatinib and nilotinib, dasatinib is active against most of the imatinib-related mutations that lead to resistance. Dasatinib binds multiple conformations of BCR-ABL [30], unlike imatinib and nilotinib [31,32]. The ability to bind both active and inactive conformations of BCR-ABL may explain its potent activity against most of the known imatinib-resistant kinase domain mutations, with the exception of T315I [33]. Dasatinib is also more potent than imatinib, with 325-fold greater *in vitro *activity against unmutated BCR-ABL [31]. The increased potency of dasatinib, combined with its ability to bind multiple conformation of BCR-ABL, produces significant efficacy in patients with CML and Ph+ ALL. The sensitivity of BCR-ABL mutants to dasatinib can be classified as sensitive (IC_50 _≤ 5 nM), intermediately sensitive (IC_50 _= 5 to 11 nM; ie, E255K/V and F317L) and insensitive (IC_50 _> 11 nM; ie, T315I) (Table 1). T315I, a contact point mutation, is insensitive to all currently approved BCR-ABL inhibitors [30,31,33-35]. P-loop mutated BCR-ABL is generally sensitive or intermediately sensitive to dasatinib, with IC_50 _values falling in the range of 1 to 11 nM [11].

Responses to dasatinib in patients with CP CML (n = 961) have been assessed by baseline mutational status [36]. Equivalent CCyR rates were noted in imatinib-resistant patients with P-loop mutations (61 of 141; 43%) and all other patients, except those with T315I and F317L mutations (140 of 336; 42%). In this study, no patients (0 of 20) with T315I mutations and only 7% (1 of 14) of patients with F317L mutations achieved CCyRs. These mutations are therefore insensitive to dasatinib. With regard to individual P-loop mutations, CCyR rates were similar to or above those of patients without mutated BCR-ABL: G250E, 37% (19 of 51); Y253F/H, 52% (12 of 23); and E255K/V, 33% (8 of 24) (Table 2). Patients with CP CML enrolled in the phase 2 START-C trial were also evaluated by baseline mutational status [37]. The results from this trial were similar to those above. One resistant patient with a Q252H mutation was observed; however, further data are needed to determine the sensitivity of this mutation to dasatinib. Moreover, as this mutation is more sensitive to dasatinib than E255K *in vitro*, it is probable that patients with Q252H mutations would respond at least as well as those with E255K/V. Based on the available data, P-loop mutations are not likely to pose a significant barrier to treatment with dasatinib.

Mutations have been shown to develop with dasatinib exposure. In an *in vitro *mutagenesis study, nine dasatinib-resistant mutations involving six residues were found. However, only F317V and T315I were isolated at intermediate drug concentrations, and T315I was the only mutation to be detected at maximal achievable plasma concentrations [38]. In clinical studies, T315A/I, F317I/L and V299L are the most frequent mutations associated with dasatinib resistance [37,39-41]. In the phase 2 START-C trial of patients with CP disease, new mutations were detected in 11% of patients (22 of 201), including 6% (13 of 201) with T315A/I, F317L or V299L (4 of 201, 7 of 201 and 2 of 201 patients, respectively) [38]. Importantly, these mutations are uncommon at baseline. Among all baseline mutations, F317L and T315I mutations have been detected with frequencies of approximately 5% each [37]. T315A and V299L mutations were not detected.

### Nilotinib

Nilotinib is an analog of imatinib with 10-fold to 50-fold greater potency against unmutated BCR-ABL than its parent compound [35]. The approval of nilotinib was based on the release of data from a single open-label phase 2 study of patients with CP or AP CML who were resistant or intolerant to prior imatinib therapy [42,43].

In the phase 2 study, following at least 6 months of treatment, rates of MCyR and CCyR rates were 48% and 31%, respectively [42]. Among patients who achieved a MCyR, 96% continued treatment without progression or death for at least 6 months (Table 3). In total, 11% of patients discontinued treatment because of disease progression in this study.

Most AEs were mild to moderate in severity and were generally able to be managed with dose reduction or interruption and appropriate supportive care. The most frequent grade 3/4 AEs in patients with CP CML were neutropenia (29%), thrombocytopenia (29%), asymptomatic serum lipase elevation (14%) and bilirubin elevation (9%) [42]. In total, 15% of patients discontinued treatment as a result of AEs [43]. Cross-intolerance was defined as the reoccurrence of a grade 3/4 AE during nilotinib treatment that caused the discontinuation of imatinib. Cross-intolerance to nilotinib occurred in 49% of patients with hematologic intolerance to imatinib, mostly due to the reoccurrence of thrombocytopenia [44]. In clinical trials, nilotinib treatment has been associated with prolongation of the QTc interval, and sudden deaths have occurred, which are likely related to ventricular repolarization abnormalities. The prescribing information for nilotinib carries a black box warning regarding the risk of these events [18].

Nilotinib has clinical activity in patients with all BCR-ABL mutations associated with imatinib resistance except T315I [42]. However, accumulated evidence suggests that nilotinib also possesses relative insensitivities to certain BCR-ABL mutations. Ten nilotinib-insensitive BCR-ABL mutations have been identified *in vitro *[38]. In contrast to dasatinib, P-loop mutations are among the most resistant, with IC_50_s ranging from 38 nM to 450 nM [11]. In a mutagenesis study, the P-loop mutations Y253H and E255V were persistent at intermediate drug concentrations and, as with dasatinib, only T315I was isolated at maximal achievable plasma concentrations [38].

In the key phase 2 study, no CCyRs were observed in patients harboring L248V, Y253H or E255K/V mutations [42]. Additionally, patients with G250E mutations had a CCyR rate of 25%, which is lower than that in the overall population (30%). In another study in patients with CP CML receiving nilotinib, no patients with F359C/V mutations experienced a CCyR [45] (Table 2). Y253H and E255K/V mutations are also among those that develop most frequently during nilotinib therapy and have been linked to disease progression [46]. Y253H, E255K/V and F359C mutations occurred in 8% of those assessed for baseline mutations (23% of all mutations). These mutations were associated with disease progression in 50% of cases [45]. Among patients with AP CML, the same mutations were associated with disease progression in 64% [47]. Notably, the incidences of nilotinib-resistant mutations at baseline and at progression are higher than those for dasatinib-resistant mutations. The P-loop mutations E255K/V, Y253H and F359C/V accounted for 25% (26 of 104) of all baseline mutations [45]. Furthermore, among 40 imatinib-resistant patients who developed mutations during nilotinib therapy, 22 (55%) had newly detectible mutations of the P-loop (10 [25%] with E255K/V; 7 [18%] with G250E; and 5 [13%] with Y253H).

### Future agents

Because none of the currently available TKIs are effective against T315I mutations, there is a clear need to develop alternative options for patients with such mutations. Several agents are in clinical development, including novel TKIs and aurora kinase inhibitors.

Bosutinib (SKI-606) is a dual Src/Abl TKI with 200-fold greater potency than imatinib against BCR-ABL in biochemical assays [48]. Bosutinib is currently being evaluated in a phase 3 trial of patients with CP CML. Unfortunately, *in vitro *studies have shown that bosutinib is not active against T315I [49,50]. In a phase 1/2 study, 48 patients with CP CML who were imatinib resistant or intolerant were treated with bosutinib 500 mg daily [51]. Of evaluable patients, 84% (16 of 19) achieved a complete hematologic response (CHR), and MCyRs were achieved in 52% (11 of 21). The most common grade 3/4 toxicities occurring in ≥ 5% of patients were thrombocytopenia (6%) and rash (6%). Diarrhea (69%), nausea (44%), vomiting (19%), abdominal pain (13%) and rash (13%) were the most common grade 1/2 toxicities. Given that bosutinib has minimal activity against c-Kit and platelet-derived growth factor receptors, it may be associated with a lower incidence of AEs related to the inhibition of these targets (eg, edema, muscle cramps, skin rash, pigmentation, endocrine abnormalities, low-grade inhibition of normal hemopoiesis) than other TKIs [49,50].

In the phase 1/2 trials of bosutinib, 13 imatinib-resistant mutations were identified in 32 patients. Preliminary results showed CHR in 12 of 14 patients with non-P-loop mutations and 3 of 3 patients with P-loop mutations. MCyR was demonstrated in 5 of 11 patients with non-P-loop mutations and 1 of 1 patient with P-loop mutations [51].

Other agents in development that may prove useful against T315I mutations include aurora kinase inhibitors. One such aurora kinase inhibitor, MK-0457, was the first agent to demonstrate clinical activity against the T315I phenotype [52]. In the study of 14 currently evaluable patients with CML, 11 had an objective (hematologic, cytogenetic and/or molecular) response, including all 9 patients with the T315I mutation [53]. Recently, however, clinical trials of MK-0457 were suspended due to cardiotoxicity concerns. Trials of other aurora kinase inhibitors, including PHA-739358 (phase 2), AP-24534 (phase 1) and XL-228 (phase 1), are ongoing. In early-stage clinical trials of PHA-739358, responses have been observed among patients with T315I mutations [54]. AP-24534 and XL-228 have demonstrated activity in cell culture and in mice bearing xenograft tumors expressing T315I BCR-ABL mutants [55,56]. A phase 1 open-label trial of XL-228 has been initiated in patients with Ph+ leukemia, and clinical trials of patients with drug-resistant CML are planned for AP-24534.

## Conclusion

P-loop mutations in the BCR-ABL gene account for nearly half of all mutations [12,13]. The mutations impart increased transformation potency with respect to other mutations and wild type BCR-ABL. Furthermore, Y253H and E255K/V are commonly present at baseline before second-line treatment.

Dasatinib and nilotinib have differential activity against certain mutations, including those of the P-loop. Clinical resistance to dasatinib has been noted for T315I and F317L mutations but not for P-loop mutations. Additionally, P-loop mutations rarely emerge during dasatinib treatment. Y253H or E255K/V are commonly associated with clinical resistance to nilotinib and can develop during treatment. Nilotinib resistance is also associated with other mutations (ie, F359 and T315I).

Based on the currently available data, dasatinib may be a suitable second-line therapy for patients resistant to imatinib and who harbor P-loop or F359 mutations, while nilotinib may be an appropriate treatment option for patients with F317L mutations. Clearly, additional treatments are needed for patients harboring T315I. Currently, such patients should be considered for allogeneic stem cell transplantation or entry into a clinical trial.

## List of abbreviations

CML: chronic myeloid leukemia; CP: chronic phase; AP: accelerated phase; BP: blast phase; TKI: tyrosine kinase inhibitor; IRIS: International Randomized Study of Interferon and STI571; CCyR: complete cytogenetic response; START: SRC/ABL Tyrosine kinase inhibition Activity: Research Trials of dasatinib; Ph+ ALL: Philadelphia chromosome-positive acute lymphoblastic leukemia; MCyR: major cytogenetic response; AE: adverse event; CHR: complete hematologic response.

## Authors' contributions

SC and DL involved in concept design, coordination, drafting and critically revising the manuscript.
